# Ten years of detecting *Neoehrlichia mikurensis* infections in Sweden: demographic, clinical and inflammatory parameters

**DOI:** 10.1007/s10096-024-04909-5

**Published:** 2024-08-13

**Authors:** Christine Wennerås, Linda Wass, Beatrice Bergström, Anna Grankvist, Christine Lingblom

**Affiliations:** 1 Department of Infectious Diseases, The Sahlgrenska Academy, University of Gothenburg, Göteborg, Sweden; 2 Department of Clinical Microbiology, Sahlgrenska University Hospital, Göteborg, Sweden; 3 Department of Clinical Immunology and Transfusion Medicine, Sahlgrenska University Hospital, Göteborg, Sweden

**Keywords:** *Neoehrlichia mikurensis*, Rituximab, Thromboembolism, Vasculitis, Tick, Blood transfusion

## Abstract

**Purpose:**

To increase knowledge about the varied clinical manifestations of human infection with the emerging tick-borne pathogen *Neoehrlichia mikurensis*.

**Methods:**

All patients diagnosed in Sweden with *N. mikurensis* infection during a 10-year period (2013–2023) were investigated regarding their demographic factors, risk factors, comorbidities, clinical signs and symptoms, and laboratory results. Multivariate models were generated using “Orthogonal Projections to Latent Structures-Discriminant Analysis” to identify clinical and immune parameters associated with *N. mikurensis* infection.

**Results:**

During the 10-year period, 134 patients were diagnosed with *N. mikurensis* infection, 102 of whom were included in this study. Most of the patients (79%) were immunosuppressed. The main comorbidities were malignant B-cell lymphomas, multiple sclerosis, and rheumatoid arthritis. Rituximab therapy (59%) and splenectomy (14%) featured prominently. All patients resided in the southern tick-endemic part of Sweden, yet one-third of them were diagnosed in wintertime when ticks are inactive. Two asymptomatically infected blood donors were identified but transfusion-transmitted infection was not confirmed. Increased levels of C-reactive protein, orosomucoid, and total IgM in serum were associated with neoehrlichiosis. Previously unreported symptoms such as ankle edema, neck pain, numbness, and sudden deafness were detected in some patients. One case of aplastic anemia partially improved after eradication of the infection.

**Conclusions:**

Neoehrlichiosis is a multi-faceted emerging infectious disease.

## Key points

Human infections with tick-borne *Neoehrlichia mikurensis* can result in asymptomatic carriage, severe disease with fever and vascular events, and a possibly causal association with a variety of hematologic conditions as well as with sudden deafness.

## Introduction

The first case reports of human infection with *Neoehrlichia* (*N.*) *mikurensis* were published in 2010 from Sweden, Switzerland, and Germany [1–3]. Despite the challenges facing researchers studying this intracellular bacterium, which does not grow outside of cell lines [4] and escapes detection by routine microbiologic diagnostic methods [5], much has been learned about this emerging infectious agent over the last decade.

*N. mikurensis* was first discovered in ticks and rodents in the Netherlands, Norway and Russia in 1999–2001 and assigned various *Ehrlichia*-based names [5]. This changed in 2004, when Japanese scientists reported that the same bacterial species had been detected in wild rodents on the Japanese island of Mikura; they showed using molecular methods that this bacterium belongs to the genus *Neoehrlichia* [6]. They named it ”*Candidatus* Neoehrlichia mikurensis” [6]; the Candidatus eponym is given to bacterial species that have not yet been isolated and cultivated. The *Neoehrlichia* genus belongs to the family *Anaplasmataceae*, which encompasses various species that are pathogenic for humans, e.g., *Anaplasma phagocytophilum* and *Ehrlichia chaffeensis.* All these pathogens are transmitted to humans primarily via tick bites.

*N. mikurensis* is harbored by ticks of the *Ixodes ricinus* complex (*Ixodes ricinus*, *Ixodes persulcatus*), and voles act as natural reservoirs [5]. It is part of the Eurasian fauna, and has been detected in ticks and/or rodents in Austria, Belgium, Bosnia-Herzegovina, Bulgaria, the Czech Republic, Denmark, Estonia, Finland, France, Germany, Hungary, Italy, The Netherlands, Moldova, Norway, Poland, Spain, Romania, Russia, Serbia, Slovakia, Sweden, Switzerland, Ukraine, China, Mongolia, South Korea and Japan [5, 7–16]. In contrast, reports of human infection with *N. mikurensis* are fewer, mainly comprising case reports [1–3, 17–23], some retrospective analyses of stored blood samples [19, 24–27], and a few prospective studies [28–34].

After the first published case reports, 9 years passed before the bacterium could be isolated from human blood and cultivated in cell lines derived from ticks and human vascular endothelium [4]. Publications after 2019 have frequently dropped the “Candidatus” designation, such that *Neoehrlichia mikurensis* has become the accepted name for this bacterium. The first whole-genome sequences of *N. mikurensis* strains isolated from patients with neoehrlichiosis were published in 2021 [35].

It is no exaggeration to claim that *N. mikurensis* infections are under-diagnosed. Currently, specific PCR tests directed against *N. mikurensis* or 16 S rRNA-based molecular methods performed on EDTA-anticoagulated blood, preferably plasma or buffy coat, are the only diagnostic tools available [35]. Another reason for the under-diagnosis of *N. mikurensis* infections is the variable clinical picture of this new infectious disease, which may include symptoms that are not commonly associated with infectious conditions, such as vascular events. The majority (60%) of a cohort of 40 Swedish patients with neoehrlichiosis had vascular and/or thromboembolic events that involved the venous or arterial blood vessels [28]. The bacteria are able to grow and propagate inside human endothelial cells in vitro, and have been detected in circulating endothelial cells isolated from the blood samples of patients with *N. mikurensis* infection [4].

The aim of this study was to characterize those patients who were diagnosed with neoehrlichiosis in Sweden during a 10-year period with regards to demographic factors (age, sex, geographic residence), risk factors (comorbidities, immune status), mode of transmission (tick exposure, blood transfusion), duration of disease prior to diagnosis, clinical picture, bacterial load, and laboratory findings. Our laboratory is the only one in Sweden to offer diagnostic PCR for this emerging infection, so this cohort is a national cohort. We carried out multivariate analyses of the data, expecting to identify a set of clinical and laboratory parameters that would facilitate the diagnosis of this new infectious disease with its diverse manifestations and expand existing knowledge of the pathogenesis of neoehrlichiosis.

## Materials and methods

### Patients

One hundred and two patients out of the 134 patients who were diagnosed at the Department of Clinical Microbiology, Sahlgrenska University Hospital, Göteborg, in the period of 2013–2023 were included in the study. Thirty-two patients were excluded from the study because they had been diagnosed through retrospective screening of stored blood samples, undergone *N. mikurensis*-screening for the purposes of other scientific studies, or had not provided written informed consent. All the patients resided in Sweden and had provided written informed consent to participate in the study, which was approved by the Regional Ethics Committee of Göteborg (Dnr 394 − 12). Clinical data were retrieved from patients’ charts and tick exposure data were obtained through a questionnaire.

### *N. mikurensis* PCR

EDTA-treated plasma was concentrated from a volume of 1.5 mL when possible. Automated DNA extraction and a Real-Time PCR directed against a 169-bp-fragment of *groEl* were performed as previously described [19].

### Statistics

The unpaired two-tailed Mann-Whitney test was used to compare two groups. A *P*-value < 0.05 was deemed to be statistically significant. “Orthogonal Projections to Latent Structures - Discriminant Analysis” (O-PLS-DA) was implemented using the SIMCA-P (ver. 15.0.2) statistical package (MKS Data Analytics Solutions, Malmö, Sweden). OPLS-DA is an extension of principal component analysis, in which Y-variables are introduced and their relationships to X-variables are evaluated. Multivariate models were created in which patients who were infected with *N. mikurensis* were set as Y-variables and 28–30 parameters (tick exposure, clinical and laboratory data) were set as X-variables. The quality levels of the models are indicated by their explanatory power, R2Y, such that a high value indicates that the X-variables have generated a model that can segregate the studied groups. The stability and robustness of the models are indicated by Q2Y, which is computed by cross-validation, whereby one study subject is removed in a stepwise manner and the capacity of the remaining subjects to predict the separation between the groups is evaluated.

## Results

**Fig. 1:**
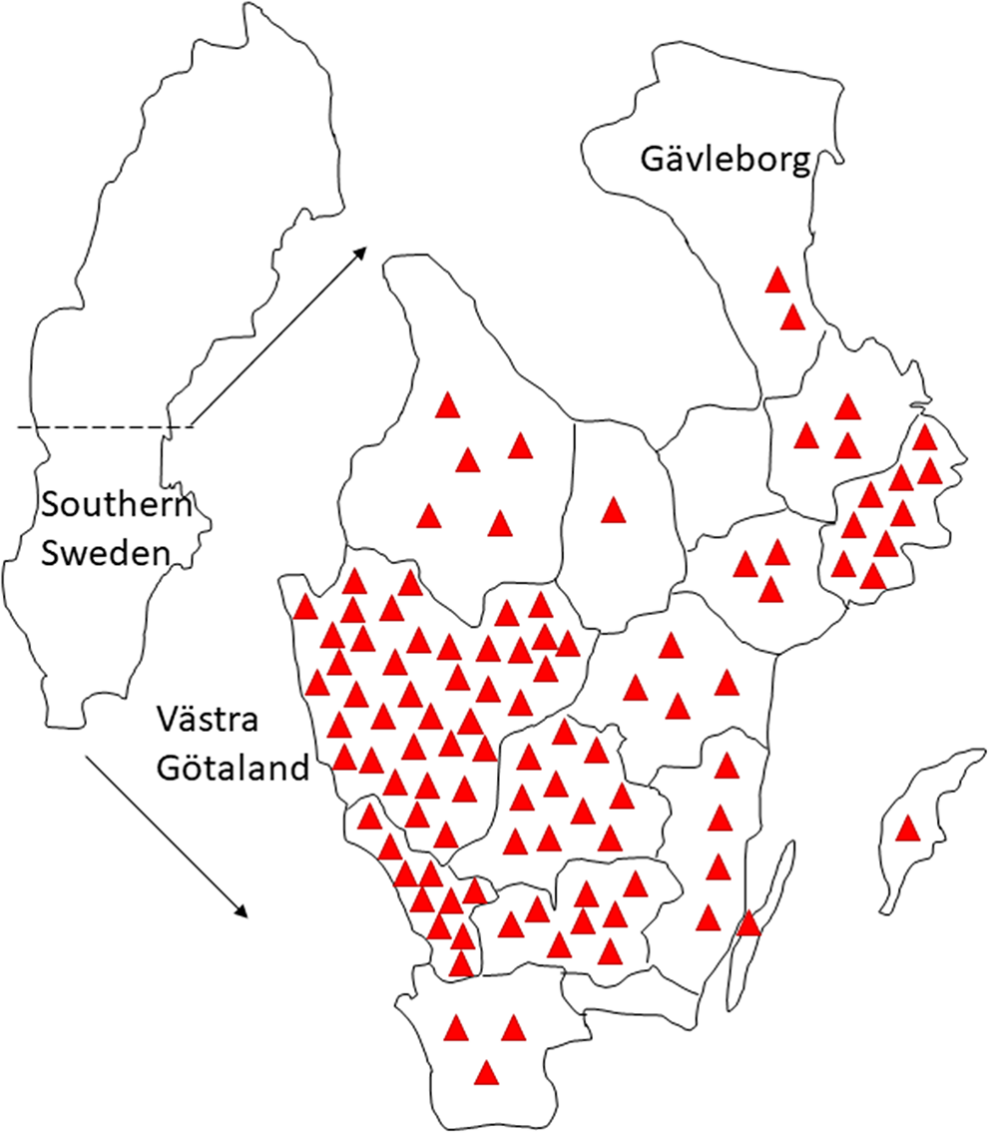
Patients with neoehrlichiosis reside in southern Sweden. The geographic distribution of the 102 study patients in the Swedish counties is shown. Each patient is indicated by a triangle

**Fig. 2:**
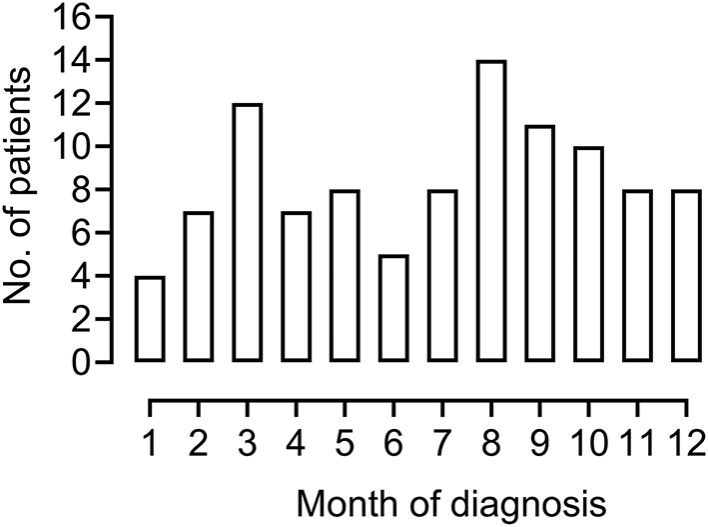
Even seasonal distribution of diagnosed cases of neoehrlichiosis. Bar graph showing the number of patients with neoehrlichiosis (*n* = 102) who were diagnosed in each month of the year (Months 1–12; period of January - December)

This cohort comprising 102 consecutive individuals diagnosed with *N. mikurensis* encompasses all evaluable patients who were diagnosed in Sweden during this 10-year period. The median age of the study group was 62 (range, 29–87) years with an even sex distribution (50% women/men). All patients resided in the southern part of Sweden; the northern limit of diagnosed cases was Gävleborg County, and the highest number of patients was diagnosed in Västra Götaland County (*n* = 38, 37%) (Fig. 1**)**. The incidence of tick exposure was high with 77/98 of the patients (78%) having experienced a tick bite at some point in their life, and with every forth tick-bitten individual reporting more than one tick bite per year. Roughly half of the patients (*n* = 57, 56%) recalled a tick bite within the last year prior to diagnosis. Patients were diagnosed with *N. mikurensis* infection regardless of the time of the year, with close to every third patient (30%) being diagnosed during the winter months of December-March when ticks are not believed to be active (maximum average temperature of + 3 °C) **(**Fig. 2**).** There was a considerable diagnostic delay before a diagnosis of *N. mikurensis* infection was established: the median duration of symptoms prior to diagnosis was 3 months (25/75 percentiles = 1–7 months), including a few cases with suspected symptom duration of several years. There were no fatalities in the cohort. All patients were treated with oral doxycycline except for a doxycycline-allergic individual who received rifampicin.

Most of the patients had compromised immunity (*n* = 81, 79%) due to an underlying hematologic, rheumatologic, neurologic, or other autoimmune condition that was being treated with immunosuppressive therapy (Table 1). More than half of the patients (59%) had ongoing treatment with the B-cell suppressive agent rituximab, making it the most frequently used immunosuppressant in this cohort of patients (Table 2). One-third of the patients, including some on rituximab therapy, received additional immunosuppressive therapy. More than one in ten patients was splenectomized (Table 2). One patient with idiopathic aplastic anemia featuring low hemoglobin levels and modest neutropenia made a partial hematologic recovery with near normalization of hemoglobin levels after the *N. mikurensis* infection was eradicated with antibiotics.

**Table 1:** Comorbidities of patients diagnosed with *N. mikurensis* infection (*n* = 102)

Disease	No. of Patients	Percent
**Rheumatologic**	24	24
Rheumatoid arthritis	13	13
Granulomatosis with polyangiitis	5	5
Lupus erythematosus	3	3
Other	3	3
**Neurologic**	24	24
Multiple sclerosis	17	17
*Myasthenia gravis*	4	4
Other	3	3
**Hematologic**	22	22
Malignant B-cell lymphoma	17	17
Other	5	5
**Gastroenterologic**	5	5
Crohn’s disease	4	4
Inflammatory bowel disease, U^a^	1	1
Hypertension	15	15
Autoimmune hemolytic anemia	3^b^	3
Sarcoidosis	4	3
Psoriasis	3	3
**None**	13	13

^a^U, unclassified, ^b^One patient had chronic lymphocytic leukemia

**Table 2:** Immune deficiencies and immunosuppressive agents for patients diagnosed with *N. mikurensis* infection (*n* = 102)

Immune status	No. of patients	Percent
*Immunosuppressed*	81	79
Rituximab treatment	60	59
Other immune-suppressive therapy^a^	33	32
Systemic corticosteroids	31	30
Splenectomy	14	14
Combined variable immune deficiency	3	3
*Immunocompetent*	21	21

^a^Including chemotherapy

The most common symptoms of neoehrlichiosis were fever, localized muscular pain, and vascular events (Table 3). More unusual symptoms, such as ankle edema, neck pain and numbness, were also reported (Table 3). Four patients experienced sudden hearing loss, one of whom had this symptom as the sole presenting sign; unfortunately, the hearing loss was permanent and did not improve after antibiotic treatment (Table 3). Two patients suffered repeated episodes of infection with *N. mikurensis* at intervals of 2 and 3 years, respectively. Both patients had ongoing rituximab therapy, and one of them reported residual pain and fatigue after completion of the first course of antibiotics.

Two asymptomatic individuals who were unknowingly infected with *N. mikurensis* (both of whom were blood donors) were part of the cohort (Table 3). A look-back investigation of the recipients of blood components from these donors could not ascertain any case of transfusion-transmitted infection with *N. mikurensis*, even though an unused platelet concentrate derived from one of the donors was PCR-positive for *N. mikurensis*. While this donor had previously donated blood to one of the study participants who was also infected with *N. mikurensis*, it could not be excluded that the recipient of the donated blood components had contracted the infection through a tick bite.

**Table 3:** Reported symptoms and clinical findings for persons diagnosed with *N. mikurensis* infection

Symptom/Clinical finding	No. of patients
Fever	84
Localized muscular pain	51
Vascular events	47
Fatigue	47
Chills	45
Sweats	29
Ankle edema/Stiff legs	20
Skin rash	16
Neck pain	10
Numbness	5
Sudden deafness	4
Asymptomatic^a^	2

^a^Healthy blood donors

**Fig. 3:**
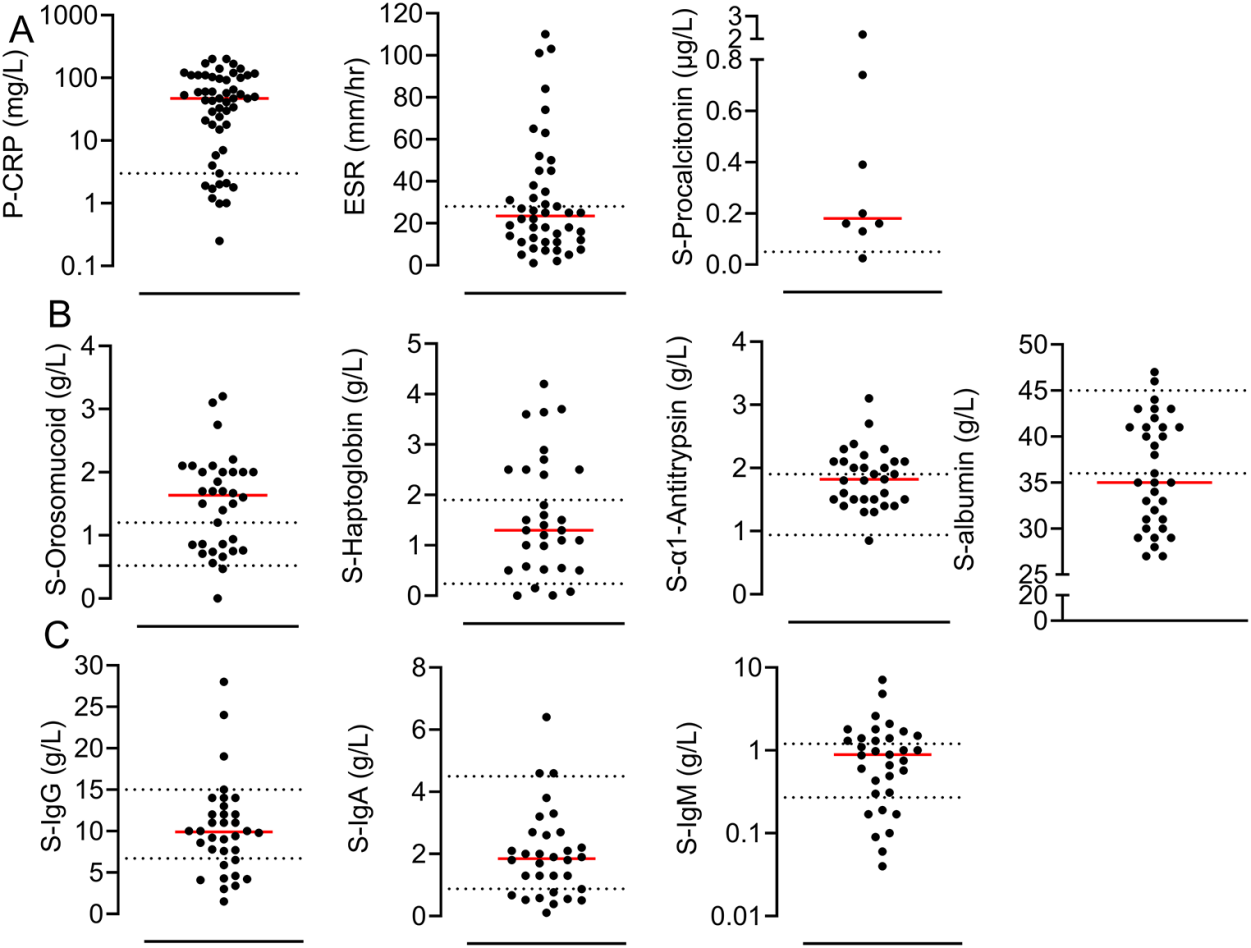
Inflammatory markers and immune parameters detected in the blood samples of patients with neoehrlichiosis. The most abnormal values were selected when repeat analyses were performed. (**A**) Serum levels of C-reactive protein (CRP) (*n* = 53), erythrocyte sedimentation rate (ESR) (*n* = 44), and concentrations of procalcitonin (*n* = 8). (**B**) Serum levels of acute-phase reactants S-orosomucoid (*n* = 34), S-haptoglobin (*n* = 31), S-α1-antitrypsin (*n* = 31), and S-albumin (*n* = 33). (**C**) Total levels of immunoglobulin G (IgG) (*n* = 34), IgA (*n* = 32), and IgM (*n* = 33) in the serum. Each dot represents a single patient. The red bar indicates the median and the dotted lines delineate the reference levels for healthy individuals

We evaluated the laboratory analytes that are part of the blood work-up for patients with suspected infections, regarding their potentials to facilitate the diagnosis of *N. mikurensis* infections. White blood cell (WBC) counts were increased (54%), normal (36%) and decreased (9%) in the evaluated patients (*n* = 52); the neutrophil counts were increased (49%), normal (42%) and decreased (8%) (*n* = 47); the lymphocyte counts were normal (41%), increased (7%) and decreased (52%) (*n* = 29); and the platelet counts were increased (21%), normal (58%), and decreased (21%) (*n* = 48). Increased levels of C-reactive protein in plasma (P-CRP) were more commonly seen than increased erythrocyte sedimentation rates (ESR) (Fig. 3a). The procalcitonin levels in serum were determined for only a few cases and were moderately increased (Fig. 3). Orosomucoid was the most discriminatory protein of the acute-phase reactants measured in the serum; the median level of S-orosomucoid was above the normal range whereas the median levels of S-α1-antitrypsin and S-haptoglobin were within the corresponding normal ranges (Fig. 3b). The median level of S-albumin was below the normal range, indicating redirection of protein production in the liver in favor of acute-phase reactants (Fig. 3b). Finally, the total serum levels of immunoglobulin G (IgG) and IgA were mostly within the normal range, as opposed to the median levels of IgM that were near the upper limit for normal serum IgM levels (Fig. 3c).

**Fig. 4:**
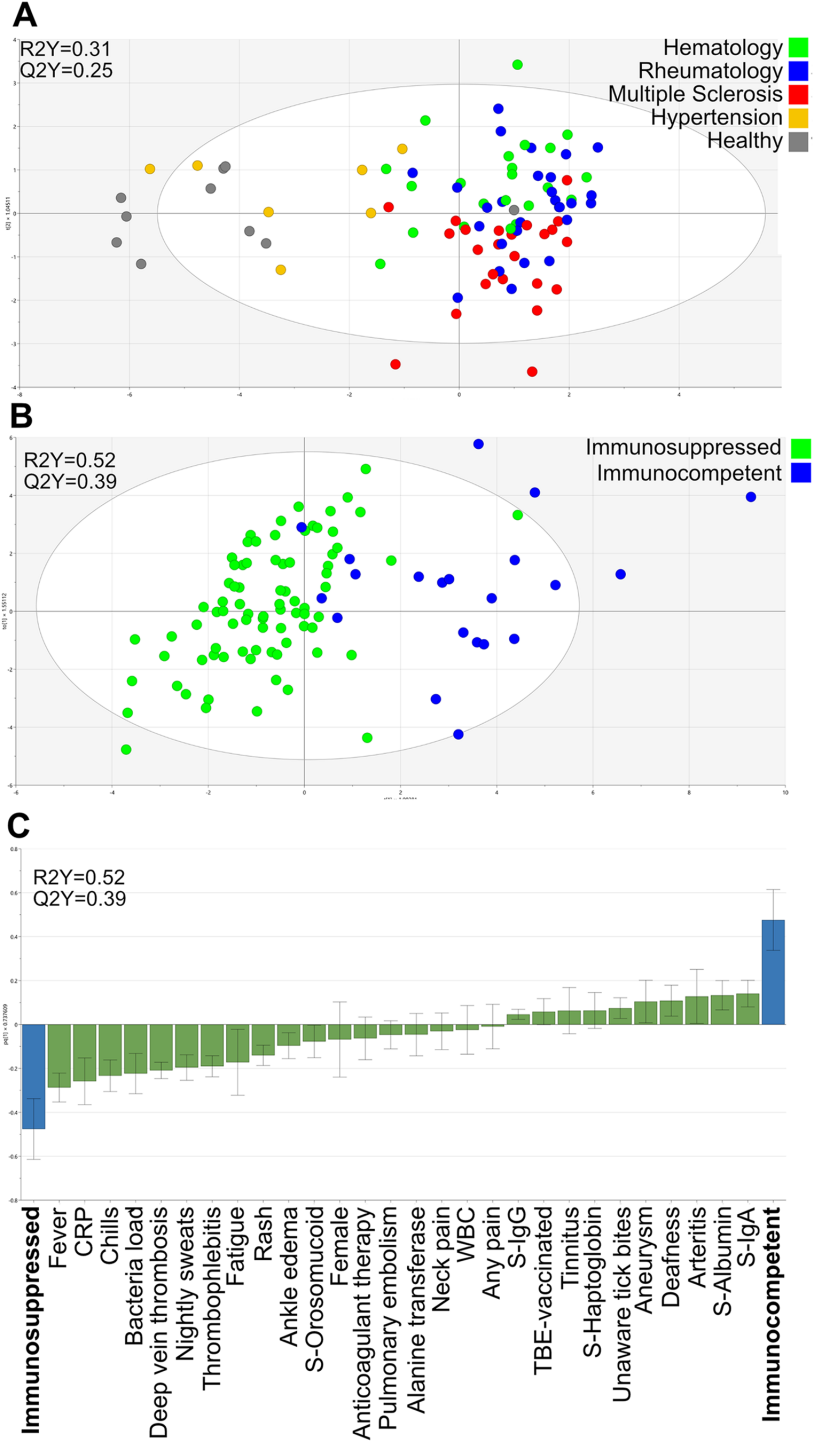
Multivariate O-PLS-DA models showing the clusters formed by **(A)** the major patient groups, i.e., patients with hematologic diseases (green), rheumatologic diseases (blue), multiple sclerosis (red), hypertension comorbidity (yellow) and healthy subjects (gray). Each dot indicates a single patient. The generated two-component model has an explanatory power of 31% (R2Y = 0.31) and stability of 25% (Q2Y = 0.25). **(B)** Immunosuppressed (green) versus immunocompetent (blue) patients. Each dot indicates a single patient. The model has an explanatory power of 52% (R2Y = 0.52) and stability of 39% (Q2Y = 0.39). **(C)** Loading plot depicting the 28 parameters (X-variables) that exert the strongest impacts on model B, in that they can segregate the infected immunosuppressed patients from the infected immunocompetent patients. The size of each parameter bar and its direction indicates its discriminatory power and positive association with the patient category bar, and its inverse relationship to the opposite patient category

We employed O-PLS-DA to determine if the patients with *N. mikurensis* infection formed clusters depending on their health status and comorbidities. The following major patient groups were compared: healthy, hypertensive comorbidity, and those with rheumatologic, hematologic, or neurologic diseases. It is clear from Fig. 4a that the healthy subjects and the hypertensive patients overlap, and are distinct from the rheumatologic, hematologic, and multiple sclerosis (MS) patients, who form an overlapping cluster with a tendency for the MS and hematologic patients to segregate within the cluster. The main difference between the healthy/hypertensive groups and the MS/rheumatologic/hematologic groups was immune status, which is why we chose to create a model that compared the infected patients with normal immunity to infected patients with compromised immune defenses. There was a near-complete separation of the immunosuppressed from the immunocompetent patients (Fig. 4b). A loading plot revealed that the parameters that were most discriminatory for the immunosuppressed patients were fever, chills, sweats, fatigue, venous vascular events (deep vein thrombosis, thrombophlebitis, pulmonary embolism), skin rash, ankle edema, and pain engaging the neck or other part of the body, all factors that were seldom reported by the patients with normal immune defenses (Fig. 4c). Instead, the immunocompetent patients more often presented with sudden deafness, tinnitus, and arterial vascular events such as arteritis and aneurysms, which rarely featured among the immunosuppressed patients (Fig. 4c).

**Fig. 5:**
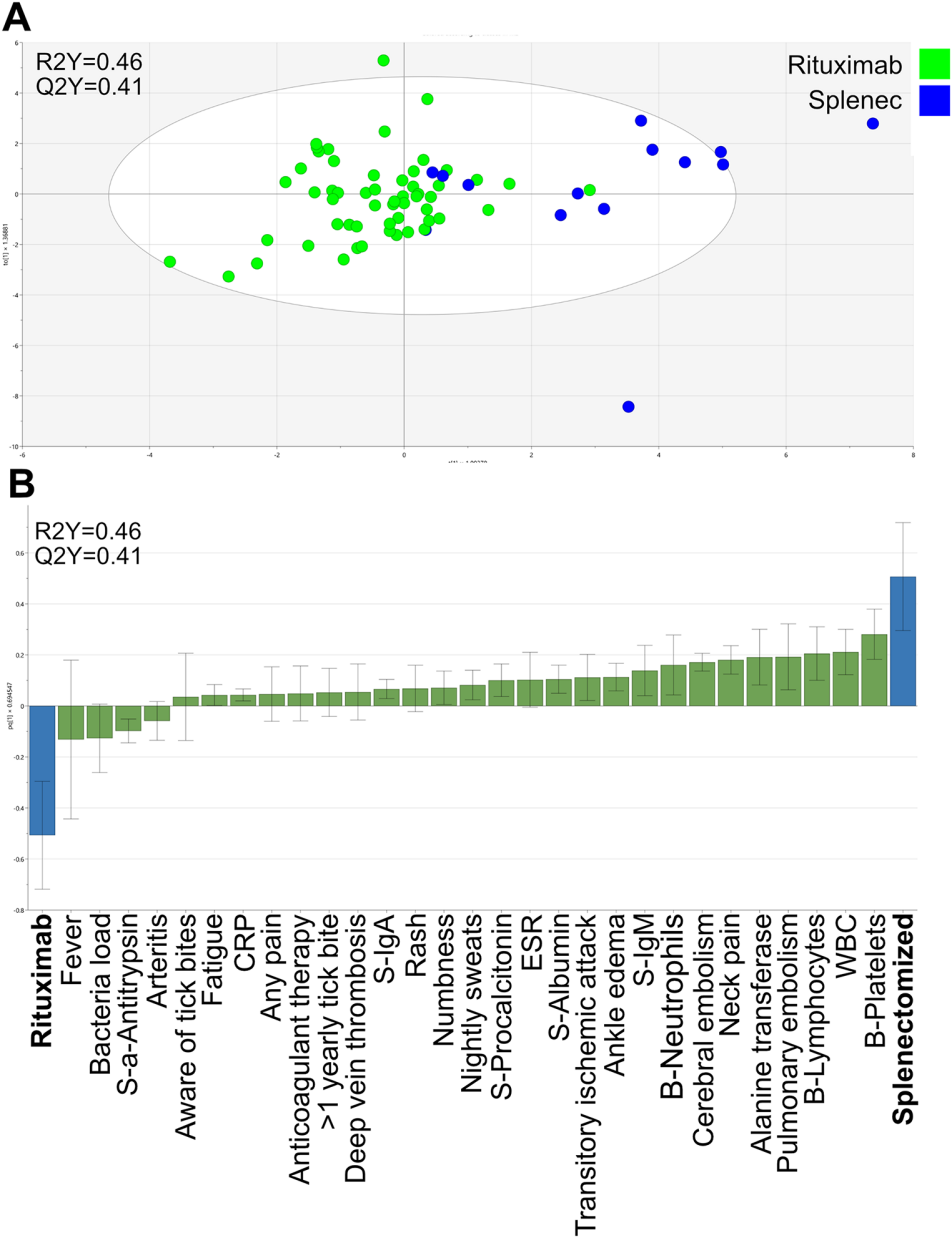
Multivariate OPLS-DA model depicting (**A**) the clusters formed by the infected patients treated with rituximab (green) and the infected splenectomized (blue) patients, respectively. Each dot indicates a single patient. The model’s explanatory power was 46% (R2Y = 0.46) and its stability was 41% (Q2Y = 0.41). (**B**). Loading plot showing the 29 parameters (X-variables) that contributed to the separation of the two groups of patients shown in **A**. The size of each parameter bar and its direction indicates its discriminatory power and positive association with the patient category bar, and its inverse relationship to the opposite patient category

A high bacterial load was one of the parameters that discriminated most clearly between the immunosuppressed and the immunocompetent patients (Fig. 4c). The cycle threshold (CT) values in the *N. mikurensis* PCR test were significantly lower, indicative of a higher bacterial load, among the immunosuppressed patients (median CT value, 24; 25–75 percentile: 20–29) compared to the immunocompetent patients (median CT value, 35; 25–75 percentile: 32–38) (*P* < 0.001 Mann-Whitney test). Higher levels of P-CRP, S-orosomucoid, WBC, and S-alanine transferase were features of the immunosuppressed, infected patients, whereas the immunocompetent, infected patients presented with higher levels of S-albumin, S-IgA, and S-IgG (Fig. 4c). Finally, the immunocompetent, infected patients often had no recall of previous tick bites, despite being more frequently vaccinated against tick-borne encephalitis virus than the immunosuppressed patients, with the latter being more frequently on anticoagulant therapy (Fig. 4c). Lastly, we compared the rituximab-treated patients with the splenectomized patients regarding clinical and immune parameters (Fig. 5a) and found that fever, increased levels of S-α1-antitrypsin, and higher bacterial loads were more frequent among the rituximab-treated patients (Fig. 5b). In contrast, the splenectomized patients tended to have higher levels of platelets and higher WBC, including lymphocytes and neutrophils, and were more frequently afflicted by pulmonary embolism, cerebral embolism, neck pain, ankle edema, transitory ischemic attacks, and deep vein thrombosis, as well as being more often in receipt of anticoagulant therapy. Moreover, the splenectomized individuals had higher levels of S-alanine transferase and total IgM in their sera, as well as higher ESRs and higher procalcitonin levels compared with the rituximab-treated patients (Fig. 5b).

## Discussion

Most of the patients in this study (80%) were aware of having been bitten by a tick. This is a surprisingly high rate of tick recall, given that other studies of tick-borne infections have reported tick recall rates in the range of 20–67% [36]. We did not question the patients regarding their history of blood transfusions, which although less likely, represents a possible means of transmission. Two of the subjects in the current study were blood donors, and *N. mikurensis* DNA was detected in a blood component (platelets) and in a recipient of one of these donors. We were unable to sequence the genomes of the two *N. mikurensis* isolates to determine if the strains from the blood donor and the recipient were identical. A study from south-eastern Sweden revealed that 0.7% of the blood donors were asymptomatically infected with *N. mikurensis* but there was no evidence of transfusion-related transmission of *N. mikurensis* infection [29]. Although it remains to be seen if blood transmission of *N. mikurensis* can occur, it is not entirely unlikely given the reported blood transmission of related bacterial species, e.g., *A. phagocytophilum* and *E. ewingii*, despite the blood products having undergone leukocyte reduction and irradiation [37, 38].

Neoehrlichiosis is likely an infectious vasculitis and the presence of inflamed and/or blocked blood vessels in various parts of the body might explain the diverse, and sometimes peculiar, symptoms displayed by infected individuals, including numbness and sudden deafness. Sudden hearing loss, which can be caused by disturbances of the blood flow to the ear, has been described in patients with vasculitis [39]. In addition, we have documented partial improvement of aplastic anemia following the elimination of *N. mikurensis* infection, which may indicate a causal relationship, as has been shown for other infectious agents, mainly viruses. Recently, a compelling case of hemophagocytic lymphohistiocytosis (HLH) in a lymphoma patient with *N. mikurensis* infection that was cured by antibiotics was reported from Denmark [40]. Again, it is mainly chronic viral infections that have been identified as causative agents of HLH. Finally, our group has identified a possible linkage between *N. mikurensis* and the development of certain types of malignant B-cell lymphomas [34]. Taken together, these findings are suggestive of associations between *N. mikurensis* infections and various hematologic conditions, and in some cases these may be causal.

In the present study, we confirm that immune status dictates the clinical picture of *N. mikurensis* infections. Thus, patients with compromised B-cell immunity have a characteristic clinical profile that features recurrent fever, chills, fatigue, thrombophlebitis, venous thromboembolic events, pain, and skin rashes that may resemble *erythema nodosum* [41]. Previous studies and the current study indicate that infected persons with normal immune defenses may instead present with little or no fever, and in severe cases, arterial vasculitis with or without tissue infarction, mimicking systemic rheumatic diseases such as polyarteritis nodosa or Takayasu arteritis [28, 31, 42]. Adding a further layer of complexity, asymptomatic carriage of the infection has been demonstrated not only in immunocompetent individuals, including blood donors, but also in immunosuppressed patients [29, 32, 33]. In addition, some of the apparently asymptomatic carriers have reported increased well-being and resolution of unspecific symptoms after eradication of the infection [32].

Thrombocytopenia, which was an infrequent finding among the patients with neoehrlichiosis, can help to distinguish this infection from anaplasmosis and spiroplasmosis [43]. Measurements of CRP and orosomucoid could also prove useful in the diagnostic work-up of patients with suspected neoehrlichiosis. The currently recommended treatment for *N. mikurensis* infection is oral delivery of 100 mg doxycycline twice daily for 3 weeks. Shorter courses of treatment or lower doses of doxycycline result in recurrence of the infection, at least in immunosuppressed individuals [40, 44]. To our surprise, two of the cases in the cohort had a repeat infection with *N. mikurensis* 2–3 years following the first infection, which raises the question as to whether the original infection had been adequately treated or whether this was really a new infection.

This national cohort of patients infected with *N. mikurensis* comprised adults only. In fact, to date, no children have been diagnosed with *N. mikurensis* infection. This is intriguing when one considers that: (1) children are frequently afflicted by *Borrelia* infections; (2) *Borrelia burgdorferi* sensu lato and *N. mikurensis* are transmitted by the same tick species [5]; and (3) these two bacterial species preferentially co-inhabit the same ticks [45]. The vascular endothelium appears to be the main target of infection for *N. mikurensis* [4]. Could it be that *N. mikurensis* is less capable of infecting the smooth, non-atherosclerotic blood vessels of children?

To conclude, our study uncovers new aspects of *N. mikurensis* infections that warrant further, in-depth exploration, including the possibility of blood transmission, the optimal duration and dosage of antibiotic treatments to eradicate the infection, the potential associations between the infection and sudden deafness, and with a variety of hematologic conditions.

## Data Availability

No datasets were generated or analysed during the current study.
